# Sympatric divergence of the ergot fungus, *Claviceps purpurea*, populations infecting agricultural and nonagricultural grasses in North America

**DOI:** 10.1002/ece3.7028

**Published:** 2020-12-12

**Authors:** Miao Liu, Parivash Shoukouhi, Kassandra R. Bisson, Stephen A. Wyka, Kirk D. Broders, Jim G. Menzies

**Affiliations:** ^1^ Ottawa Research and Development Centre Agriculture and Agri‐Food Canada Ottawa ON Canada; ^2^ Colorado State University Fort Collins CO USA; ^3^ Smithsonian Tropical Research Institute Panama Panama; ^4^ Morden Research and Development Centre Agriculture and Agri‐Food Canada Morden MB Canada

**Keywords:** Ascomycota, house‐keeping gene, multilocus haplotype, neutrality, phylogenetic network, population structure, selective sweeping

## Abstract

The ergot diseases of agricultural and nonagricultural grasses are caused by the infection of *Claviceps* spp. (Hypocreales, Ascomycota) on florets, producing dark spur‐like sclerotia on spikes that are toxic to humans and animals, leading to detrimental impacts on agriculture and economy due to the downgrading of cereal grains, import–export barriers, reduced yield, and ecological concerns. At least seven phylogenetic lineages (phylogenetic species) were identified within the premolecular concept of *C. purpurea* s.l. (sensu lato) in agricultural areas and vicinities in Canada and the Western United States. *Claviceps purpurea* s.s (sensu stricto) remained as the most prevalent species with a wide host range, including cereal crops, native, invasive, and weedy grasses. The knowledge on genetic diversity and distribution of *C. purpurea* s.s. in North America is lacking. The objective of the present study was to shed light on genetic differentiation and evolution of the natural populations of *C. purpurea* s.s. Multilocus DNA sequences of samples from Canada and the Western USA were analyzed using a phylogenetic network approach, and population demographic parameters were investigated. Results showed that three distinct genetically subdivided populations exist, and the subdivision is not correlated with geographic or host differentiations. Potential intrinsic mechanisms that might play roles in leading to the cessation of gene flows among the subpopulations, that is, mating and/or vegetative incompatibility, genomic adaptation, were discussed. The neutrality of two house‐keeping genes that are widely used for DNA barcoding, that is, translation elongation factor 1‐α (*TEF*1‐α) and RNA polymerase II second largest subunit (*RPB2*), was challenged and discussed.

## Graphical abstract

Regardless of the frequent sexuality and multiple modes of dispersal, the population of the toxigenic plant pathogen, *Claviceps purpurea*, that infects wide range of agricultural and nonagricultural grasses, was subdivided in nature. The population structure shed light on the underline mechanisms that might have led to the cessation of gene flow among subdivided populations. House‐keeping gene markers were not neutral in certain circumstances.

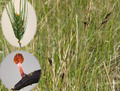

## 1 INTRODUCTION

**FIGURE 1:**
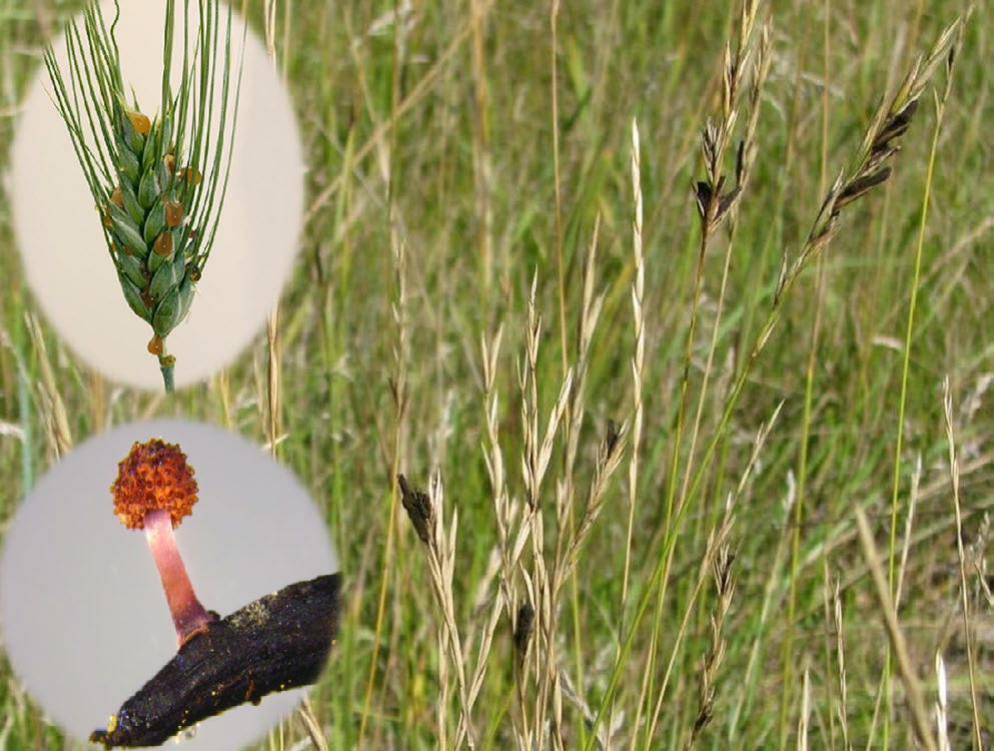
*Claviceps purpurea* sclerotia on grasses *Elymus repens* in the field heads (background), the asexual stage in honeydew after inoculating a barley plant in greenhouse (upper inset), and the sexual stage produced from a germinating sclerotium in a controlled environment (bottom inset)

Elucidating the genetic structure of plant pathogen populations that infect both agricultural and nonagricultural host populations can provide insight into the evolutionary history of the pathogen populations, and can be useful for predicting the potential development of new races, effective population size, dispersal potential, and the probability for host range expansion or the emergence of more virulent races of the pathogen. The ergot diseases of cereal crops, forage grasses, native, and invasive grasses are caused by the infection of *Claviceps* spp. (Hypocreales, Ascomycota) on florets, producing dark spur‐like sclerotia (Figure 1) on spikes that are toxic to humans and animals. During the last ten years, the incidence and severity of ergot in agricultural crops (barley, rye, wheat) as well as forage, native, and weedy grasses has increased in the eastern and prairies provinces of Canada (Menzies & Turkington, 2015; Xue et al., 2017) as well as regions of the western United States including barley growing regions in Colorado, Montana, and Wyoming (Wyka, 2020).

The broad host range of the premolecular *C. purpurea* s.l. (sensu lato), including more than 400 species of Poaceae (Alderman et al., 2004; Campbell 1957; Píchová et al. 2018), and cosmopolitan distribution suggested a potential species complex that was later proven with evidence pointing toward adaptation to ecological niches (Douhan et al. 2008; Liu et al., 2020; Pazoutova et al. 2000, 2002, Pažoutová et al., 2015; Shoukouhi et al., 2019). At least seven phylogenetic lineages (phylogenetic species) were identified within *C. purpurea* s.l. across Canada and the United States, including *C. purpurea* s.s. (sensu stricto), *C. humidiphila, C. occidentalis*, *C. perihumidiphila, C. quebecensis, C. ripicola,* and *C. spartinae* (Liu et al., 2020; Shoukouhi et al., 2019). *Claviceps purpurea* s.s. was the predominant species recovered, with a wide host range including cereal crops and forage grasses, comprising 90% of samples collected (data not shown). Host specificity studies under controlled conditions showed virulence variations between the *C. purpurea* s.l. isolates (Cagaš & Macháč, 2002; Menzies et al., 2017), suggesting genetic variation between isolates. Previous pathogenicity and population studies of *C. purpurea* likely included multiple species within *C. purpurea* s.l. and therefore may not accurately represent the population dynamics of this fungus (*C. purpurea* s.s., Cagaš & Macháč, 2002; Campbell, 1957; Gilmore et al., 2016).

During an annual life cycle of *C. purpurea* s.l, both sexual and asexual propagules are produced and cause infections. The primary infection occurs in spring or early summer by windborne ascospores (sexual) released from ascostromata developed from overwintered sclerotia. While the infection of late flowering plants is primarily caused by secondary inocula in the form of asexual conidia, which is immersed in honeydew that oozes from florets and is transmitted by insect vectors, rain splash, or direct head‐to‐head contacts (Figure 1; Campbell & Freisen, 1959; Tenberge, 2006). Global commercialization of the seeds contaminated with ergots (sclerotia) can lead to human‐mediated long‐distance dispersal (Munkvold, 2009). The annual sexual reproduction within the population likely results in genetic recombination among the strains in the field, increasing genotypic diversity. However, this would also homogenize populations preventing population differentiation. Meanwhile, the abundance and polycyclic nature of asexual secondary propagules may contribute to shaping clonal population structures (Milgroom, 2015c). The observed fluctuation of disease incidence in western Canada and the United States may reflect a population bottleneck and expansion causing genetic drift that could have also impacted the population structure (Menzies et al., 2017). To elucidate which forces have impacted on the evolution of this fungus in nature, an insight to the population structure is imperative.

Multilocus genotyping data combined with population network analyses can be used to explore genetic differentiation and evolution of natural populations. Phylogenetic network analyses are suitable for reticulate relationships caused by various population processes, that is, recombination, gene conversion, lineage sorting, and deep coalescence (Posada & Crandall, 2001). This provides a better inference of the relationships among populations than strict phylogenetic analyses assuming a bifurcate evolutionary pattern (Bapteste et al., 2013; Morrison, 2005). Population demographic parameters reflect the signatures of the natural forces that have shaped the population's structure (as reviewed by Charlesworth & Charlesworth, 2017). The main objective of this study was to investigate the population structure of the ergot fungus*, C. purpurea* s.s in Canada and Western USA to determine whether the population represents a single panmixia or subdivide to several populations, and whether or not these subdivisions are influenced by geography and/or host association.

## 2 MATERIALS AND METHODS

### 2.1 Fungal isolates and DNA sequences

Floret samples infected by *Claviceps purpurea* were collected from agricultural areas and vicinities in Canadian provinces and Western United States: Alberta (AB), Manitoba (MB), Ontario (ON), Quebec (QC), Saskatchewan (SK), and Colorado (CO), supplemented with occasional samples from British Columbia (BC), Montana (MT), Newfoundland (NL), Nova Scotia (NS), and Wyoming (WY). Sclerotia from the same host and location (<100 m^2^) were pooled as one sample, as such a total of 303 samples were obtained. Axenic fungal cultures were isolated and purified for 252 samples; other 51 samples were as sclerotia without pure culture (collected from ON and QC in 2016, also see the Table S1). For genomic DNA (gDNA) extraction, a small portion of mycelia from axenic cultures (252 samples) was plucked or a fraction of the sclerotia (51 samples) was taken after surface sterilization. A high‐throughput protocol was used on a KingFisher Flex magnetic particle processor (Thermo Fisher Scientific Oy) with Macherey‐Nagel NucleoMag^®^ 96 Trace kit (Machery Nagel GmbH & Co. KG) following the manufacturer's manual. All fungal isolates, sources (culture or sclerotium), hosts, and locations were provided in Table S1. All gDNA samples were subjected to PCR for four gene regions: RNA polymerase II second largest subunit (*RPB2*) using *Claviceps* specific forward primer TTTCGTGGTATTGTTCGCAGA (Pažoutová et al., 2015) and fRPB2‐7cR (Liu et al., 1999), translation elongation factor 1‐α (*TEF*1‐α) using EF1‐983F and EF1‐2218R (Pažoutová et al., 2015; Rehner & Buckley, 2005), ergot alkaloid chanoclavine I synthase oxidoreductase (*easE*) using easE996f and easE1895r, and ergot alkaloid chanoclavine I aldehyde oxidoreductase (*easA*) using easA547f and easA867r (Shoukouhi et al., 2019). PCR and sequencing followed the protocols developed by Shoukouhi et al. (2019). Twenty‐four reference sequences of 14 related species were downloaded from GenBank to be used for confirming the identities of all 303 samples (Table S1).

### 2.2 Data analysis

The DNA sequences for each gene were aligned using online version MAFFT (Katoh et al., 2017), accessed on 02‐02‐2020 with auto strategy (FFT‐NS‐1, FFT‐NS‐2, FFT‐NS‐i or L‐INS‐i; depends on data size). The resulting alignments were eye‐adjusted: Big gaps at both ends due to unequal length of sequences were removed by shortening alignments; short indels (1–2 nts) in the middle due to polymers were adjusted so that the coding regions can be properly assigned (all the gene regions amplified are exons). The alignments of four genes were concatenated using Geneious Prime v.2020.1.2 (https://www.geneious.com), and missing loci were treated as gaps. To confirm the identities of all samples, the concatenated alignment was appended with the 24 reference sequences of 14 related species, and subjected to a phylogenetic analysis using PAUP* 4.0b10 (Swofford, 2002). The most parsimonious trees were searched for using heuristic branch‐swapping algorithm, tree‐bisection‐reconnection (TBR), 100 replicates, number of rearrangements per replicate limit 5,000, and bootstrap replicates 2000.

A subset of samples that have been sequenced for all four genes was submitted to population demographic analyses and network analyses as follows. The analyses of DNA polymorphism, nucleotide diversity, haplotype diversity, and neutrality were performed using dnaSP v6.10.04 (Rozas et al., 2017) for the individual genes and concatenated matrices. Testing for neutrality was evaluated using Tajima's D with total number of mutation (Tajima, 1989), Fu, and Li's D* and F*(Fu & Li, 1993). Haplotypes were generated and analyzed for each gene and concatenated alignment.

Phylogenetic network analyses were conducted for the haplotypes of concatenated DNA sequences using SplitsTree4 V4.14.8 (Huson & Bryant, 2006). A neighbor‐net method with four different variance calculations was tested, that is, ordinary least square, FitchMargoliash1, FitchMargoliash2, and Estimated variance. The clustering patterns were further tested using BEAST2 v2.5 (Bayesian evolutionary analysis sampling trees) with a multilocus coalescent model (Bouckaert et al., 2019), which estimates rooted, time‐measured phylogenies. The best‐fit models for each partition genes were selected by Akaike information criterion (AIC) or hierarchical likelihood ratio tests (hLRTs) through Modeltest 3.7 (Posada & Crandall, 1998), that is, GTR or TrN for *TEF*1‐α, K81 + I+G for *RPB*2, TVMef + I+G or K80 + I+G for *easE*, and SYM + I or K80 + G for *easA*. Only four substitute model options (JC69, HKY, TN93, and GTR) were available in BEASTv2.5, and therefore, we set GTR for *TEF*1‐α, and HKY for the other three genes (HKY was considered as an extension of K80, and K81 models). Other priors were set as default, 10,000,000 generations, sampling frequency 1,000, burn‐in 10%, and link trees. Resulting phylogenies were visualized using DensiTree v 2.0.0 (Bouckaert & Heled, 2014). To further test the evolutionary trajectories inferred by BEAST, we performed phylogenetic analyses for the haplotypes aligned with closely related species and out‐groups using PAUP* 4.0b10. The heuristic search protocols were the same as described earlier.

Genetic differentiation between subpopulations (genetic clusters, abbreviated as GC in the following text, tables and figures) was further tested by the analysis of molecular variance (AMOVA, with 9,999 permutations) and the principle coordinate analysis (PCoA) in GenAlEx 6.5 (Peakall & Smouse, 2012). The haplotype‐SNP matrix from the concatenated alignment was used to generate a pairwise individual‐by‐individual (N × N) genetic distance matrix, which was used for subsequent calculation of Φ_PT_ (analogous of *F*_ST_) via AMOVA between subpopulations (genetic clusters), PCoA, Mantel, and spacial autocorrelation analyses.

The sequence‐based statistics, *S*_nn_ measuring the frequency in which the “nearest‐neighbor” sequences or haplotypes belong to the same subpopulation, were considered suitable for both high haplotype diversity and low haplotype diversity (Hudson, 2000). *S*_nn_ and gene flow parameter, *N*_m_, the migration number per generation (Nei, 1982) was estimated in DnaSp v6.

**FIGURE 2:**
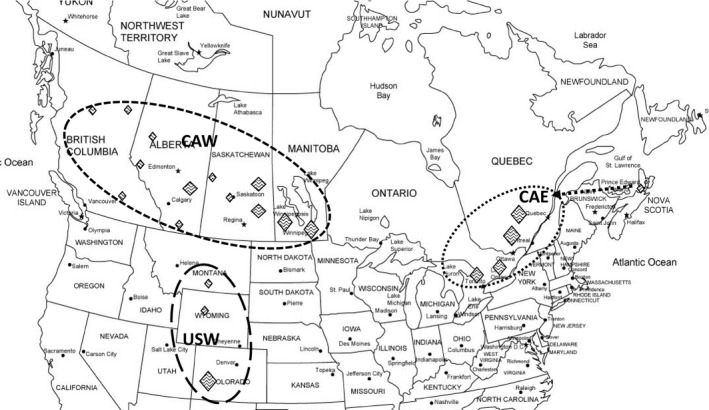
Geographic locations of studied samples, and designation of geographic regions: western Canada (CAW), eastern Canada (CAE) and western USA (USW). Larger sizes of rhombus marks indicate more samples

Next, we investigated whether the genetic differentiation revealed by phylogenetic networks and statistic tests were correlated with geographic separation, or host ranges as follows. Based on geographic location, 156 samples were separated into three geographically separated populations: 1. Western Canada (AB, BC, MB, SK); 2. Eastern Canada (ON, QC, NL, NS); and 3. Western US (CO, MT, WY) (Table 1, Figure 2). We examined the differentiation among three geographic regions by genotypic components, AMOVA, PCoA, and pairwise genetic differentiation, Φ_PT_ statistics, and compared with the estimates for genetic clusters. To test for isolation by distance, we conducted Mantel tests to understand the correlation between genetic distances and geographic distances. For calculating geographic distance, the approximate XY coordinates were obtained from converting the center of the named locations (town/city/agricultural district, Table 1). For a few haplotypes shared by multiple locations, the medians of coordinates were used. All these tests were performed in GenAlEx 6.5.

**TABLE 1:** 146 haplotypes of concatenated sequences from four loci (153 SNPs), frequency, isolate collecting information, and subpopulation assignments

Haplotype	Fr^a^	Isolate	Host	Host Tribe	Year	Country, Province (State)	Location	Approximate Coordinates^b^	Genetic Cluster^c^	Geographic population^c^	Host goup^c^
Hap1	1	Clav01	*Elymus trachycalus*	*Triticeae*	2016	US, CO	Saguache, Center	37.751584, −106.111029	GC3	USW	Tri
Hap2	1	Clav02	*Bromus inermis*	*Bromeae*	2016	US, CO	Saguache, Center	37.751584, −106.111029	GC3	USW	Bro
Hap3	1	Clav03	*Bromus inermis*	*Bromeae*	2016	US, CO	Saguache, Center	37.751584, −106.111029	GC3	USW	Bro
Hap4	1	Clav04	*Bromus inermis*	*Bromeae*	2016	US, CO	Saguache, Center	37.751584, −106.111029	GC3	USW	Bro
Hap5	1	Clav05	Poaceae	n.a.	2016	US, CO	Saguache, Center	37.751584, −106.111029	GC3	USW	n.a.
Hap6	1	Clav06	*Thinopyrum intermedium*	*Triticeae*	2016	US, CO	Saguache, Center	37.751584, −106.111029	GC3	USW	Tri
Hap7	1	Clav08	*Bromus inermis*	*Bromeae*	2016	US, CO	Rio Grande, Del Norte	37.679591, −106.355537	GC3	USW	Bro
Hap8	1	Clav09x1	*Bromus inermis*	*Bromeae*	2016	US, CO	Saguache, Center	37.751584, −106.111029	GC3	USW	Bro
Hap9	2	Clav09x2	*Bromus inermis*	*Bromeae*	2016	US, CO	Saguache, Center	37.751584, −106.111029	GC3	USW	Bro
		Clav09x3	*Bromus inermis*	*Bromeae*	2016	US, CO	Saguache, Center	37.751584, −106.111029	GC3	USW	Bro
Hap10	1	Clav10	*Achnatherum robustum*	*Stipeae*	2016	US, CO	Rio Grande, Monte Vista	37.579963, −106.151511	GC3	USW	n.a.
Hap11	1	Clav11	*Pascopyrum smithii*	*Triticeae*	2016	US, CO	Saguache, Center	37.751584, −106.111029	GC1	USW	Tri
Hap12	1	Clav12	*Bromus inermis*	*Bromeae*	2016	US, CO	Rio Grande, Del Norte	37.679591, −106.355537	GC2	USW	Bro
Hap13	1	Clav13	*Bromus inermis*	*Bromeae*	2016	US, CO	Saguache, Center	37.751584, −106.111029	GC1	USW	Bro
Hap14	1	Clav14	*Achnatherum robustum*	*Stipeae*	2015	US, CO	San Luis Valley	37.725066, −105.851177	GC2	USW	n.a.
Hap15	1	Clav16	Poaceae	n.a.	*n*.*n*.	US, CO	Saguache	37.751584, −106.111029	GC3	USW	n.a.
Hap16	1	Clav18	*Hordeum vulgare*	*Triticeae*	2015	US, CO	Rio Grande, Monte Vista	37.579963, −106.151511	GC3	USW	TriC
Hap17	1	Clav19	*Hordeum vulgare*	*Triticeae*	2015	US, CO	Rio Grande, Del Norte	37.679591, −106.355537	GC3	USW	TriC
Hap18	1	Clav20	*Hordeum vulgare*	*Triticeae*	2015	US, CO	Rio Grande, Monte Vista	37.579963, −106.151511	GC3	USW	TriC
Hap19	1	Clav21	*Hordeum vulgare*	*Triticeae*	2015	US, CO	Rio Grande, Monte Vista	37.579963, −106.151511	GC3	USW	TriC
Hap20	1	Clav23	*Hordeum vulgare*	*Triticeae*	2015	US, CO	Saguache, Center	37.751584, −106.111029	GC1	USW	TriC
Hap21	1	Clav25	*Hordeum vulgare*	*Triticeae*	2015	US, CO	Saguache	37.751584, −106.111029	GC3	USW	TriC
Hap22	1	Clav26	*Hordeum vulgare*	*Triticeae*	2015	US, CO	Saguache, Center	37.751584, −106.111029	GC3	USW	TriC
Hap23	1	Clav27	*Hordeum vulgare*	*Triticeae*	2015	US, CO	Rio Grande, Monte Vista	37.579963, −106.151511	GC2	USW	TriC
Hap24	1	Clav28	*Hordeum vulgare*	*Triticeae*	2015	US, CO	Saguache, Center	37.751584, −106.111029	GC3	USW	TriC
Hap25	1	Clav29	*Hordeum vulgare*	*Triticeae*	2016	US, WY	Washakie, Worland	44.016538, −107.957727	GC3	USW	TriC
Hap26	1	Clav33	*Hordeum vulgare*	*Triticeae*	2015	US, MT	Yellowstone, Huntley	45.902089, −108.306116	GC1	USW	TriC
Hap27	1	Clav37	*Achnatherum robustum*	*Stipeae*	2017	US, CO	Rio Grande, Monte Vista	37.579963, −106.151511	GC1	USW	n.a.
Hap28	1	Clav38	*Bromus inermis*	*Bromeae*	2017	US, CO	Alamosa	37.470983, −105.878860	GC2	USW	Bro
Hap29	1	Clav39	*Bromus inermis*	*Bromeae*	2017	US, CO	Rio Grande, Monte Vista	37.579963, −106.151511	GC1	USW	Bro
Hap30	1	Clav40	*Thinopyrum intermedium*	*Triticeae*	2017	US, CO	Rio Grande, Monte Vista	37.579963, −106.151511	GC3	USW	Tri
Hap31	1	Clav41	*Phleum pratense*	*Poeae*	2017	US, CO	Saguache, Center	37.751584, −106.111029	GC3	USW	n.a.
Hap32	1	Clav42	*Phleum pratense*	*Poeae*	2017	US, CO	Rio Grande, Del Norte	37.679591, −106.355537	GC3	USW	n.a.
Hap33	1	Clav43	*Sporobolus airoides*	*Zoysieae*	2017	US, CO	Saguache, Center	37.751584, −106.111029	GC3	USW	n.a.
Hap34	1	Clav44	*Bromus inermis*	*Bromeae*	2016	US, WY	Washakie, Worland	44.016538, −107.957727	GC3	USW	Bro
Hap35	1	Clav45	*Bromus inermis*	*Bromeae*	2016	US, WY	Washakie, Worland	44.016538, −107.957727	GC3	USW	Bro
Hap36	1	Clav46	*Secale cereale*	*Triticeae*	2016	US, WY	Washakie, Worland	44.016538, −107.957727	GC2	USW	TriC
Hap37	1	LM1000	*Ammophila breviligulata*	*Poeae*	2018	CA, QC	Saint‐Henri‐de‐Taillon	48.679321, −71.882174	GC3	CAE	n.a.
Hap38	1	LM1004	*Ammophila breviligulata*	*Poeae*	2018	CA, QC	Saint‐Henri‐de‐Taillon	48.679321, −71.882174	GC2	CAE	n.a.
Hap39	1	LM1006	*Elymus repens*	*Triticeae*	2018	CA, QC	Saint‐Henri‐de‐Taillon	48.679321, −71.882174	GC1	CAE	Tri
Hap40	1	LM1007	*Elymus repens*	*Triticeae*	2018	CA, QC	Saint‐Henri‐de‐Taillon	48.679321, −71.882174	GC2	CAE	Tri
Hap41	1	LM1015	*Elymus repens*	*Triticeae*	2018	CA, QC	South of Lac‐Saint‐Jean	48.427272, −71.920971	GC2	CAE	Tri
Hap42	1	LM1016	*Elymus repens*	*Triticeae*	2018	CA, QC	Parc National Pointe‐Taillon	48.686714, −71.869392	GC1	CAE	Tri
Hap43	1	LM1018	*Elymus repens*	*Triticeae*	2018	CA, QC	Parc National de la Maurice	46.770014, −72.954005	GC3	CAE	Tri
Hap44	1	LM1023	*Elymus repens*	*Triticeae*	2018	CA, NS	Whycocomagh	45.973787, −61.122525	GC3	CAE	Tri
Hap45	1	LM13	*Hordeum vulgare*	*Triticeae*	1996	CA, SK	Crop District: SK5	50.766959, −102.095529	GC3	CAW	TriC
Hap46	1	LM15	*Hordeum vulgare*	*Triticeae*	1996	CA, MB	Crop District: MB7	49.958573, −98.474904	GC1	CAW	TriC
Hap47	1	LM16	*Hordeum vulgare*	*Triticeae*	1996	CA, AB	Crop District: AB5	52.319928, −114.335955	GC2	CAW	TriC
Hap48	2	LM18	*Hordeum vulgare*	*Triticeae*	1997	CA, MB	Crop District: MB7	49.958573, −98.474905	GC1	CAW	TriC
		LM20	*Hordeum vulgare*	*Triticeae*	1997	CA, MB	Crop District: MB4	51.181872, −101.294412	GC1	CAW	TriC
Hap49	1	LM19	*Hordeum vulgare*	*Triticeae*	1997	CA, SK	Crop District: SK8	52.733575, −104.079084	GC1	CAW	TriC
Hap50	2	LM206	*Bromus riparius*	*Bromeae*	2014	CA, MB	Minnewasta Golf Course	49.187079, −98.129535	GC3	CAW	Bro
		LM685	*Elymus repens*	*Triticeae*	2017	CA, ON	Ottawa, Constance bay	45.492377, −76.075568	GC3	CAE	Tri
Hap51	1	LM208	*Elymus repens*	*Triticeae*	2014	CA, MB	Morden, 59 Fairway Dr	49.187175, −98.134208	GC3	CAW	Tri
Hap52	2	LM209	*Elymus repens*	*Triticeae*	2014	CA, MB	Morden, 59 Fairway Dr	49.187175, −98.134208	GC3	CAW	Tri
		LM210	*Elymus repens*	*Triticeae*	2014	CA, MB	Minnewasta Golf Course	49.187079, −98.129535	GC3	CAW	Tri
Hap53	1	LM211	*Elymus repens*	*Triticeae*	2014	CA, MB	Minnewasta Golf Course	49.187079, −98.129535	GC2	CAW	Tri
Hap54	1	LM212	*Elymus repens*	*Triticeae*	2014	CA, MB	Minnewasta Golf Course	49.187079, −98.129535	GC3	CAW	Tri
Hap55	1	LM213	*Elymus repens*	*Triticeae*	2014	CA, MB	Minnewasta Golf Course	49.187079, −98.129535	GC2	CAW	Tri
Hap56	1	LM214	*Elymus repens*	*Triticeae*	2014	CA, MB	Minnewasta Golf Course	49.187079, −98.129535	GC1	CAW	Tri
Hap57	1	LM216	*Elymus repens*	*Triticeae*	2014	CA, MB	AAFC Morden RDC	49.184991, −98.091762	GC1	CAW	Tri
Hap58	1	LM217	*Elymus repens*	*Triticeae*	2014	CA, MB	AAFC Morden RDC	49.184991, −98.091762	GC2	CAW	Tri
Hap59	1	LM221	*Phalaris arudinacea*	*Poeae*	2014	CA, MB	Snowflake	49.063439, −98.652897	GC1	CAW	n.a.
Hap60	1	LM222	*Phalaris arudinacea*	*Poeae*	2014	CA, MB	Snowflake	49.063439, −98.652897	GC1	CAW	n.a.
Hap61	3	LM223	*Bromus riparius*	*Bromeae*	2014	CA, MB	Snowflake	49.063439, −98.652897	GC3	CAW	Bro
		LM226	*Bromus riparius*	*Bromeae*	2014	CA, MB	Snowflake	49.063439, −98.652897	GC3	CAW	Bro
		LM227	*Bromus riparius*	*Bromeae*	2014	CA, MB	Snowflake	49.063439, −98.652897	GC3	CAW	Bro
Hap62	1	LM225	*Bromus riparius*	*Bromeae*	2014	CA, MB	Snowflake	49.063439, −98.652897	GC2	CAW	Bro
Hap63	1	LM232	*Phalaris canariensis*	*Poeae*	2014	CA, MB	Oakbank, Garven Rd	49.975124, −96.975761	GC3	CAW	n.a.
Hap64	1	LM236	Ornamental grass	n.a.	2014	CA, BC	Peachland	49.778671, −119.735858	GC1	CAW	n.a.
Hap65	1	LM26	*Triticum aestivum*	*Triticeae*	2000	CA, MB	Crop District: MB7	49.958573, −98.474906	GC1	CAW	TriC
Hap66	1	LM3	*Triticale* sp.	*Triticeae*	1996	CA, MB	University of Manitoba	49.807388, −97.137305	GC2	CAW	TriC
Hap67	1	LM302	*Secale cereale*	*Triticeae*	2013	CA, MB	Brandon	49.839271, −99.930058	GC3	CAW	TriC
Hap68	1	LM326	*Bromus riparius*	*Bromeae*	2014	CA, MB	Minnewasta Golf Course	49.187079, −98.129535	GC2	CAW	Bro
Hap69	1	LM328	*Elymus repens*	*Triticeae*	2014	CA, MB	Minnewasta Golf Course	49.187079, −98.129535	GC1	CAW	Tri
Hap70	1	LM330	*Elymus repens*	*Triticeae*	2014	CA, MB	AAFC Morden RDC	49.184991, −98.091762	GC1	CAW	Tri
Hap71	1	LM331	*Elymus repens*	*Triticeae*	2014	CA, MB	Snowflake	49.063439, −98.652897	GC3	CAW	Tri
Hap72	1	LM332	*Elymus repens*	*Triticeae*	2014	CA, MB	Snowflake	49.063439, −98.652897	GC3	CAW	Tri
Hap73	1	LM335	*Phalaris canariensis*	*Poeae*	2014	CA, MB	Peachland	49.778671, −119.735858	GC3	CAW	n.a.
Hap74	1	LM35	*Triticum aestivum*	*Triticeae*	2000	CA, MB	Crop District: MB1	49.362259, −100.252217	GC1	CAW	TriC
Hap75	1	LM36	*Triticum aestivum*	*Triticeae*	2000	CA, MB	Crop District: MB3	50.322365, −100.410408	GC3	CAW	TriC
Hap76	1	LM363	*Elymus repens*	*Triticeae*	2015	CA, SK	near Wapella	50.261835, −101.972356	GC3	CAW	Tri
Hap77	1	LM372	*Elymus repens*	*Triticeae*	2014	CA, MB	Morden	49.196098, −98.106165	GC1	CAW	Tri
Hap78	1	LM382	*Bromus inermis*	*Bromeae*	2015	CA, SK	Wapella	50.261835, −101.972356	GC3	CAW	Bro
Hap79	1	LM39	*Triticum durum*	*Triticeae*	2000	CA, SK	Crop District: SK1b	50.046218, −102.230159	GC1	CAW	TriC
Hap80	2	LM399	*Bromus inermis*	*Bromeae*	2015	CA, SK	Wapella	50.261835, −101.972356	GC3	CAW	Bro
		LM406	*Bromus inermis*	*Bromeae*	2015	CA, SK	Wapella	50.261835, −101.972356	GC3	CAW	Bro
Hap81	1	LM402	*Bromus inermis*	*Bromeae*	2015	CA, SK	Wapella	50.261835, −101.972356	GC2	CAW	Bro
Hap82	1	LM403	*Bromus inermis*	*Bromeae*	2015	CA, SK	Wapella	50.261835, −101.972356	GC1	CAW	Bro
Hap83	1	LM407	*Elymus repens*	*Triticeae*	2015	CA, SK	Wapella	50.261835, −101.972356	GC1	CAW	Tri
Hap84	1	LM410	*Bromus inermis*	*Bromeae*	2015	CA, SK	Wapella	50.261835, −101.972356	GC1	CAW	Bro
Hap85	1	LM411	*Bromus inermis*	*Bromeae*	2015	CA, SK	Wapella	50.261835, −101.972356	GC2	CAW	Bro
Hap86	1	LM414	*Secale cereale*	*Triticeae*	2014	CA, SK	Scott	52.366225, −108.830474	GC3	CAW	TriC
Hap87	1	LM415	*Secale cereale*	*Triticeae*	2014	CA, SK	Watrous	51.686058, −105.466174	GC3	CAW	TriC
Hap88	1	LM420	*Bromus inermis*	*Bromeae*	2015	CA, SK	Wapella	50.261835, −101.972356	GC3	CAW	Bro
Hap89	1	LM459	*Elymus* sp.	*Triticeae*	2016	CA, ON	Nepean, Dealership Drive	45.264625, −75.782265	GC2	CAE	Tri
Hap90	1	LM46	*Triticum durum*	*Triticeae*	2000	CA, AB	Crop District: AB1	50.263277, −110.561590	GC2	CAW	TriC
Hap91	1	LM460	*Bromus inermis*	*Bromeae*	2016	CA, QC	Gatineau, Rue Notre‐Dame‐de‐l'île	45.432596, −75.710750	GC2	CAE	Bro
Hap92	1	LM463	*Elymus repens*	*Triticeae*	2016	CA, ON	St.Isidore, Caledonia Road	45.388675, −74.907616	GC1	CAE	Tri
Hap93	1	LM465	*Elymus repens*	*Triticeae*	2016	CA, ON	Ottawa, Albert Road	45.414394, −75.710383	GC1	CAE	Tri
Hap94	1	LM466	*Elymus repens*	*Triticeae*	2016	CA, ON	Casselman, Rue Principle (Route 7)	45.313275, −75.090372	GC1	CAE	Tri
Hap95	1	LM467	*Secale cereale*	*Triticeae*	2015	CA, SK	Yorkton	51.216752, −102.466594	GC1	CAW	TriC
Hap96	1	LM470	*Elymus repens*	*Triticeae*	2016	CA, ON	Ottawa, Central Experimental Farm	45.379765, −75.717087	GC2	CAE	Tri
Hap97	1	LM471	*Elymus repens*	*Triticeae*	2016	CA, ON	Ottawa, Central Experimental Farm	45.379765, −75.717087	GC1	CAE	Tri
Hap98	1	LM472	*Triticum aestivum*	*Triticeae*	2016	CA, ON	Ottawa, Central Experimental Farm	45.383782, −75.718501	GC3	CAE	TriC
Hap99	1	LM473	*Triticum aestivum*	*Triticeae*	2016	CA, ON	Ottawa, Central Experimental Farm	45.379765, −75.717087	GC3	CAE	TriC
Hap100	1	LM474	*Hordeum vulgare*	*Triticeae*	2016	CA, ON	Ottawa, Central Experimental Farm	45.383249, −75.710674	GC3	CAE	TriC
Hap101	1	LM475	*Elymus repens*	*Triticeae*	2016	CA, ON	Ottawa, Central Experimental Farm	45.388133, −75.703122	GC1	CAE	Tri
Hap102	2	LM476	*Bromus inermis*	*Bromeae*	2016	CA, ON	Ottawa, Central Experimental Farm	45.388133, −75.703122	GC2	CAE	Bro
		LM55	*Secale cereale*	*Triticeae*	2000	CA, AB	Crop District: AB4	50.960038, −110.887276	GC2	CAW	TriC
Hap103	1	LM477	*Elymus repens*	*Triticeae*	2016	CA, ON	Nepean, Barrhaven	45.264095, −75.780920	GC2	CAE	Tri
Hap104	1	LM478	*Elymus repens*	*Triticeae*	2016	CA, ON	Nepean, Barrhaven	45.264095, −75.780920	GC3	CAE	Tri
Hap105	1	LM479	Cyperaceae	n.a.	2016	CA, ON	Lansdowne, Island Parkway	44.349027, −76.094653	GC3	CAE	n.a.
Hap106	1	LM480	*Elymus repens*	*Triticeae*	2016	CA, ON	Grand Valley	43.867404, −80.307495	GC1	CAE	Tri
Hap107	1	LM481	*Elymus repens*	*Triticeae*	2016	CA, ON	*n*.*n*.	43.943560, −80.358900	GC1	CAE	Tri
Hap108	1	LM50	*Triticum durum*	*Triticeae*	2000	CA, AB	Crop District: AB2	49.958935, −112.804169	GC3	CAW	TriC
Hap109	1	LM54	*Secale cereale*	*Triticeae*	2000	CA, AB	Crop District: AB4	50.960038, −110.887276	GC2	CAW	TriC
Hap110	1	LM558	*Elymus repens*	*Triticeae*	2016	CA, QC	Saint‐Lin‐Laurentides	45.857732, −73.798360	GC1	CAE	Tri
Hap111	1	LM562	*Elymus repens*	*Triticeae*	2016	CA, QC	Maskinongé	46.228879, −73.012555	GC3	CAE	Tri
Hap112	1	LM566	*Hordeum vulgare*	*Triticeae*	2016	CA, QC	Ste‐Cécile‐de‐Milton	45.491011, −72.761878	GC1	CAE	TriC
Hap113	1	LM569	*Triticum aestivum*	*Triticeae*	2016	CA, QC	Louisville	46.258056, −73.008924	GC1	CAE	TriC
Hap114	1	LM59	*Avena sativa*	*Aveneae*	2005	CA, MB	Portage la prairie	49.969321, −98.289172	GC3	CAW	n.a.
Hap115	2	LM60	*Avena sativa*	*Aveneae*	2005	CA, MB	Portage la prairie	49.969321, −98.289172	GC3	CAW	n.a.
		LM683	*Bromus inermis*	*Bromeae*	2017	CA, ON	High Lonesome Nature Reserve	45.331743, −76.369610	GC3	CAE	Bro
Hap116	1	LM634	*Elymus repens*	*Triticeae*	2017	CA, ON	Nepean, Barrhaven	45.270924, −75.775472	GC3	CAE	Tri
Hap117	1	LM635	*Elymus repens*	*Triticeae*	2017	CA, ON	Nepean, Barrhaven	45.270290, −75.777285	GC1	CAE	Tri
Hap118	1	LM636	*Elymus repens*	*Triticeae*	2017	CA, ON	Nepean, Barrhaven	45.271271, −75.777757	GC1	CAE	Tri
Hap119	1	LM637	*Bromus inermis*	*Bromeae*	2017	CA, ON	Nepean, Barrhaven	45.271656, −75.776952	GC1	CAE	Bro
Hap120	1	LM638	*Elymus repens*	*Triticeae*	2017	CA, ON	Nepean, Barrhaven	45.271656, −75.776952	GC3	CAE	Tri
Hap121	1	LM639	*Elymus repens*	*Triticeae*	2017	CA, ON	Nepean, Barrhaven	45.271656, −75.776952	GC1	CAE	Tri
Hap122	1	LM640	*Calamagrostis xacutiflora*	*Poeae*	2017	CA, QC	Gatineau	45.432596, −75.710750	GC1	CAE	n.a.
Hap123	1	LM641	*Elymus repens*	*Triticeae*	2017	CA, ON	Mer Bleue, Dolman Ridge Rd	45.40639, −75.51833	GC1	CAE	Tri
Hap124	2	LM642	*Elymus repens*	*Triticeae*	2017	CA, ON	Woodlawn, Torbolton Ridge Rd	45.425462, −76.081984	GC1	CAE	Tri
		LM652	*Elymus repens*	*Triticeae*	2017	CA, ON	Woodlawn, Torbolton Ridge Rd	45.425462, −76.081984	GC1	CAE	Tri
Hap125	1	LM643	*Elymus repens*	*Triticeae*	2017	CA, ON	Mer Bleue, Dolman Ridge Rd	45.40639, −75.51833	GC3	CAE	Tri
Hap126	1	LM646	*Elymus repens*	*Triticeae*	2017	CA, ON	High Lonesome Nature Reserve	45.3327, −76.372	GC3	CAE	Tri
Hap127	1	LM647	*Bromus inermis*	*Bromeae*	2017	CA, ON	High Lonesome Nature Reserve	45.331743, −76.369612	GC1	CAE	Bro
Hap128	1	LM648	*Elymus repens*	*Triticeae*	2017	CA, ON	High Lonesome Nature Reserve	45.331743, −76.369613	GC1	CAE	Tri
Hap129	1	LM649	*Phleum pratense*	*Poeae*	2017	CA, ON	High Lonesome Nature Reserve	45.331743, −76.369614	GC1	CAE	n.a.
Hap130	1	LM651	*Elymus virginicus*	*Triticeae*	2017	CA, ON	Woodlawn, Torbolton Ridge Rd	45.425462, −76.081984	GC2	CAE	Tri
Hap131	1	LM653	*Elymus repens*	*Triticeae*	2017	CA, ON	Ottawa, Constance bay	45.492377, −76.075568	GC1	CAE	Tri
Hap132	1	LM655	*Pascopyrum smithii*	*Triticeae*	2017	CA, MB	AAFC Brandon RDC	49.839271, −99.930058	GC3	CAW	Tri
Hap133	1	LM656	*Elymus lanceolatus*	*Triticeae*	2017	CA, MB	AAFC Brandon RDC	49.839271, −99.930058	GC1	CAW	Tri
Hap134	1	LM657	*Elymus innovatus*	*Triticeae*	2017	CA, AB	Jasper National Park	52.869451, −118.076100	GC1	CAW	Tri
Hap135	1	LM66	Poaceae	n.a.	1996	CA, MB	Crop District: MB3	50.322365, −100.410408	GC2	CAW	n.a.
Hap136	1	LM681	*Elymus repens*	*Triticeae*	2017	CA, ON	High Lonesome Nature Reserve	45.331743, −76.369611	GC3	CAE	Tri
Hap137	1	LM686	*Pascopyrum smithii*	*Triticeae*	2017	CA, MB	AAFC Brandon RDC	49.839271, −99.930058	GC2	CAW	Tri
Hap138	1	LM710	*Lolium aruninaceum*	*Poeae*	2017	CA, ON	Ottawa, Quyon Ferry Landing	45.511693, −76.222032	GC1	CAE	n.a.
Hap139	1	LM711	*Bromus inermis*	*Bromeae*	2017	CA, BC	Peace River District	56.875329, −123.298317	GC2	CAW	Bro
Hap140	1	LM712	*Bromus inermis*	*Bromeae*	2017	CA, MB	Morden	49.196098, −98.106165	GC3	CAW	Bro
Hap141	1	LM714	*Elymus repens*	*Triticeae*	2017	CA, QC	Gatineau Park	45.583699, −75.896328	GC2	CAE	Tri
Hap142	1	LM715	*Elymus repens*	*Triticeae*	2017	CA, ON	Arnprior	45.435753, −76.351986	GC3	CAE	Tri
Hap143	1	LM717	*Elymus repens*	*Triticeae*	2017	CA, ON	Ottawa	45.422550, −75.531216	GC3	CAE	Tri
Hap144	1	LM83	*Bromus ciliatus*	*Bromeae*	1956	CA, BC	Baldonnel	56.217560, −120.689544	GC1	CAW	Bro
Hap145	1	LM88	*Secale cereale*	*Triticeae*	2015	CA, SK	near Arborfield	53.104120, −103.660996	GC1	CAW	TriC
Hap146	1	LM9	*Triticum aestivum*	*Triticeae*	1996	CA, MB	Crop District: MB7	49.958573, −98.474907	GC1	CAW	TriC

^a^ Frequency of haplotypes.

^b^ The approximate coordinates were obtained by locating the centre of the described location on Google Map. For hap50, hap52, hap102, and hap115, the medians of coordinates from multiple locations were used.

^c^ Genetic clusters: GC1—3; geographic regions: Eastern Canada (CAE), Western Canada (CAW), Western USA (USW); host groups: crop Triticeae (TriC), noncrop Triticeae (Tri), Bromeae (Bro), n.a., not applied.

Besides geographic isolation, host specialization is considered another major force driving population subdivision in plant pathogen populations (Milgroom, 2015a). The correlations between genetic clusters with three major host groups were examined: (a) Bromeae (*Bromus*, 32 haplotypes); (b) Noncrop Triticeae (*Elymus*, *Pascopyrum*, *Thinopyrum*, 56 haplotypes), and (c) Crop Triticeae (*Hordeum*, *Secale*, *Triticum*, *Trirticale*, 36 haplotypes). The two haplotypes on *Avena* (Aveneae) and other 22 haplotypes were not included in the analyses because their host species belonged to distant taxa and small sample size. Genetic differentiation among these three host groups was examined through AMOVA, PCoA, and pairwise genetic differentiation, Φ_PT_ statistics, and compared with the estimates from genetic clusters.

To understand the characteristics of each subpopulations, the allelic pattern was examined in GenAlEx through the parameters: *N*_a_ (number of different alleles), *N*_e_ (number of effective alleles = $1/(\sum P_{i}^{2})$, I (Shannon's information index = −1* ∑ (*P*_i_ * Ln (*P*_i_))), h (haploid genetic diversity = $1‐\sum P_{i}^{2}$), where *P*_i_ is the frequency of the *i*th allele for the population; $\sum P_{i}^{2}$ is the sum of the squared population allele frequencies. Nucleotide diversity (*π*), mutation rate (*θ*), recombination parameter (*R*), linkage disequilibrium, and neutrality were tested in DnaSP v6. The recombination parameters include R per gene (*R*_g,_ recombination rate per generation between the most distant sites), R per adjacent sites (*R*_a_), and minimum recombination events (*R*_m_). The parameter for linkage disequilibrium was the Kelly's Z_nS_ statistic measuring the overall association between polymorphic sites (Kelly, 1997), the average *r* ^2^ of all pairwise comparison (Hill & Robertson, 1968), Rozas' Z_a_ (association between adjacent polymorphic sites), and ZZ (= Z_nS_ − Z_a_) (Rozas et al., 2001). We compared the estimated recombination and linkage disequilibrium from each GCs with the combined populations. The rationale is that a higher level of recombination and lower level of LD is expected in subpopulations (genetic clusters) than in the combined population if the population was subdivided. Neutrality tests were conducted using Tajima's D, Fu, and Li's D* and F* to find any evidence of selection in each GC.

## 3 RESULTS

### 3.1 DNA sequences for each locus

Varied number of sequences was obtained from each locus: 227 for *TEF*1, 204 for *RPB*2, 264 for *easE*, and 274 for *easA*, among which 156 samples were successfully amplified for all four genes. All sequences were submitted to GenBank (see Data Accessibility, Table S1). The alignment of each gene resulted in matrices: *TEF*1‐α 648 sites with 227 sequences, *RPB2* 693 sites with 204 sequences, *easE* 756 sites with 264 sequences, and *easA* 246 sites with 274 sequences. The matrix of concatenated sequences of four genes along with reference sequences composed of 327 sequences and 2,345 characters. The most parsimonious trees showed that 303 samples grouped with ex‐neotype of *C. purpurea* s.s. (DAOMC 251723 = CCC771) as a clade with a 94% bootstrapping support (Figure S1) with predominant internal branches having supports lower than 70%, indicating the 303 samples belonged to a single species *C. purpurea* s.s.

Analyses of DNA polymorphism showed that *easE* had a much higher proportion of variable sites (0.127), haplotype, and nucleotide diversities (0.950 ± 0.008, 0.0144 ± 0.00023), than three other genes. *TEF*1‐α was the least variable (Table 2). For neutrality tests, *easA* showed nonsignificant deviation from neutrality based on all parameters (Tajima's D, Fu & Li's D*, Fu & Li's F*); *easE* showed nonsignificant deviation based on Tajima's D, however, significant at 0.02 critical level based on Fu & Li' *D* and *F*; *TEF*1‐α and *RPB*2 showed significant departure from neutral based on all parameters at various critical levels (*p*‐value < .01, .02, .05). Overall, Fu & Li's statistics based on coalescence simulation, as expected, showed more sensitive than Tajima's D (Table 2). In general, *easA* appeared neutral, *easE* under slight selection which can only be detected by Fu & Li's statistics, but *TEF*1‐α and *RPB*2 were likely under selection, which could be a result of highly structured population or selective sweep (see more results for tests in subpopulations and in Section 4).

**TABLE 2:** DNA polymorphism and neutrality of each loci

Loci	Number of sequences	DNA polymorphism										Average number of nucleotide differences	Neutrality					
		Site^a^	Variable sites	Ratio of variable sites	Singleton	Parsimony informative	Number of haplotypes	Haplotype diversity	Nucleotide diversity	Theta (per site)from S	Theta (per sequence) from S		Tajima's D	*p*‐value	Fu & Li's D*	*p*‐value	Fu & Li's F*	*p*‐value
	*N*	TS	S	S/TS			h	Hd ± *SD*	Pi ± *SD*	θ‐W	Θ‐W	k	TjD	P	FuLi D*	P	FuLi F*	P
*TEF1*	227	648	37	0.0571	23	14	31	0.74 ± 0.027	0.0024 ± 0.00021	0.010	6.17	1.54	−2.146	<.01 ***	−5.835	<.02^***^	−5.122	<.02^***^
*RPB2*	204	693	43	0.062	26	17	44	0.82 ± 0.019	0.0030 ± 0.00018	0.011	7.30	2.04	−2.179	<.01 ***	−6.142	<.02^***^	−5.311	<.02^***^
*easE*	264	756	96	0.127	39	57	94	0.95 ± 0.008	0.0144 ± 0.00023	0.021	15.61	10.92	−1.112	>.10 n.s.	−5.015	<.02^***^	−3.750	<.02^***^
*easA*	274	246	19	0.0772	2	17	28	0.91 ± 0.007	0.0119 ± 0.00042	0.012	3.07	2.92	−0.251	>.10 n.s.	0.131	>.10 n.s.	−0.027	>.10n.s.

a *** significant at indicated level, i.e. .01, or .02.

### 3.2 Haplotype analyses

Haplotype diversity varied between genes with the *TEF*1‐α having 31 haplotypes from 227 isolates, *RPB*2 44 haplotypes from 204 isolates, *easE* 94 haplotypes from 264 isolates, and *easA* 28 haplotypes from 274 isolates. A greater percentage of private haplotypes were identified in the first three genes, that is, 58% (18/31), 66% (29/44), and 62% (58/94), while relatively fewer private haplotypes 32% (9/28) were observed for *easA* (Table S2). For the concatenated alignment of 156 isolates, 146 haplotypes were identified, among which one was shared by three isolates, eight haplotypes consisted of 2 isolates, and all other haplotypes (94%) were unique. Of the nine shared haplotypes, only three (hap 50, hap 102, hap 115) were found in more than one region (Western Canada, Eastern Canada; Table 1).

### 3.3 Population structure analyses

**FIGURE 3:**
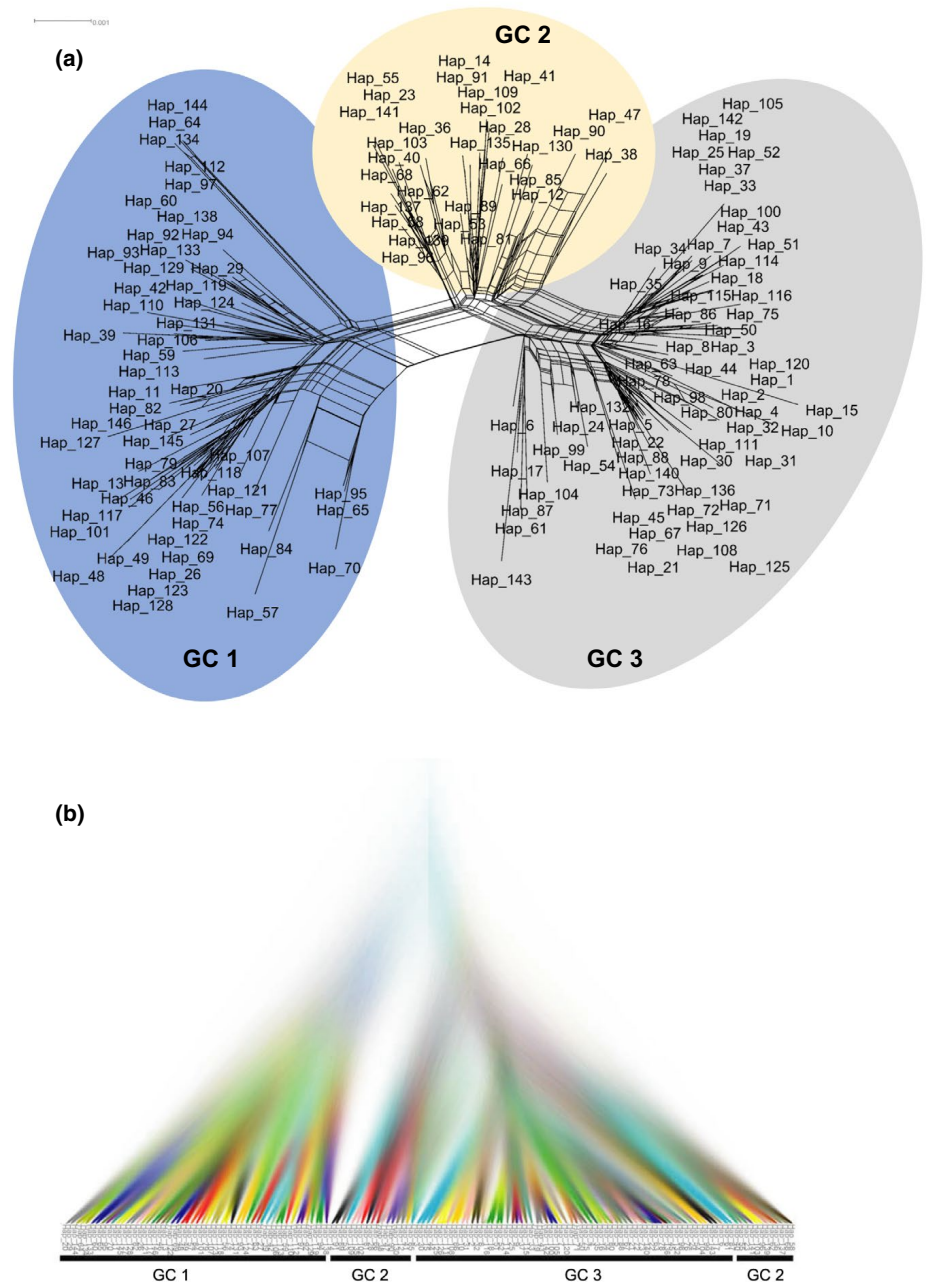
Network analyses based on 146 haplotypes of four‐locus concatenated sequences using SplitsTree4 V4.14.8 (a) and BEAST v2.5 (b)

**FIGURE 4:**
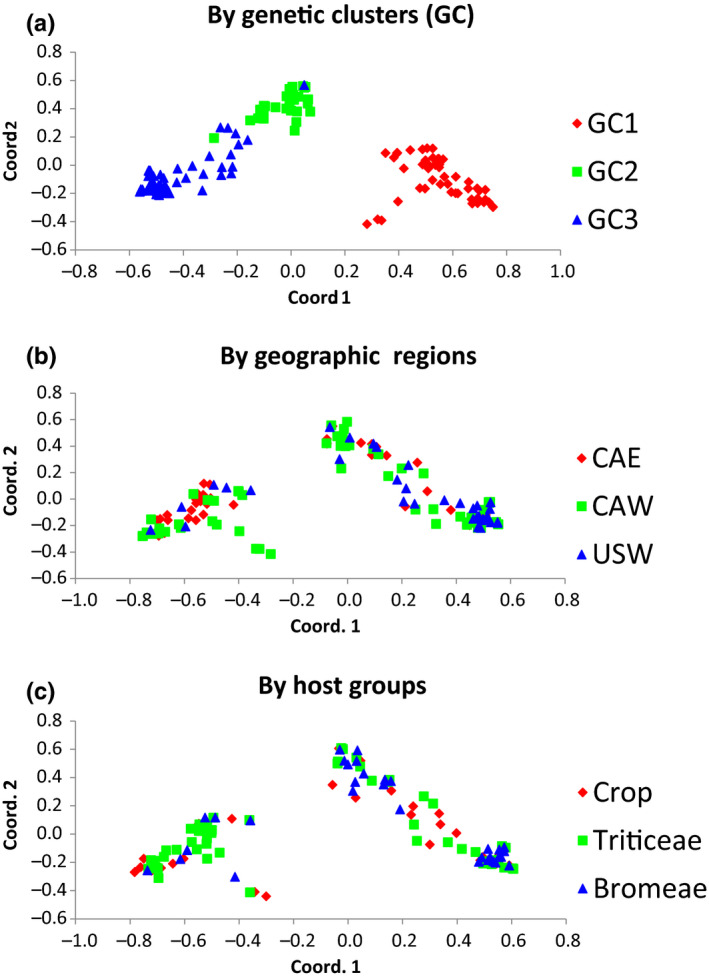
Principal coordinate analyses (PcoA) of 146 haplotypes using GenAlex 6.5. Populations were assigned based on genetic cluster (a), geographical regions (b), and host groups (c)

Three genetically distinct clusters, GC1–3, were recovered on the network generated by SplitsTree4 V4.14.8, with GC1 being more distantly related to the other two clusters, GC2 and 3 (Figure 3a). DensiTree view of the 9,001 resulting trees from BEAST confirmed the network pattern in that GC1 was clearly separated from others, while GC3 was nested inside of GC2. The branching pattern also suggested that the divergence between GC1 and the other two clusters was more ancestral, while GC3 from GC2 was more recent (Figure 3b). The rooted phylogeny of haplotypes supported this trajectory in that GC1 appeared as a paraphyletic group in relationship to GC2 and GC3, while majority GC2 samples formed a group paraphyletic to GC3 except three haplotypes located inside of GC3 clade (Figure S2). The nonreciprocal and incomplete separations of GCs indicate either recent divergence (intraspecific), or/and the lower inference power of the strict phylogenetic approach compared with networking analyses at intraspecies level. The separation of three genetic clusters was also observed in the PCoA analyses showing GC1 clearly separated from other samples, while the separation between GC2 and GC3 was not clear‐cut (Figure 4a).

Genetic differentiation among the genetic clusters was statistically significant (Φ_PH_ = 0.445, *S*_nn_ = 0.991), with very limited gene flow (*N*_m_ = 0.46; Table 3). Populations of *C. purpurea* s.s. were also significantly differentiated based on geographic regions using the permutation tests; however, *S*_nn_ (0.5808) was close to 0.5 and the PCoA did not ascertain geographic separation, indicating subpopulations were not strongly differentiated and likely belong to the same population (Hudson, 2000). In addition, molecular variance within geographic regions (96%) was much higher than that among the regions (4%). The measure of population subdivision (Φ_PH_), was much lower (0.036 versus 0.445), and gene flow level (*N*_m_ = 6.27) was much higher than among three genetic clusters. The differentiation among three host groups was not statistically significant and had a higher level of gene flow (*N*_m_ = 9.31; Table 3). PCoA analyses did not separate geographic regions or host groups (Figure 4b,c).

**TABLE 3:** Analysis of molecular variance and sequences‐based statistics for genetic clusters, geographic populations, and host groups

Parameters^d^	Genetic clusters^a^		Geographic regions^b^		Host groups^c^	
		*p*‐value		*p*‐value		*p*‐value
Molecular variance among populations	45%		4%		1%	
Molecular variance within populations	55%		96%		99%	
Φ_PT_	0.445	.001***	0.036	.003**	0.011	.106 n.s.
*S*_nn_	0.99145	.0000***	0.5808	.000***	0.376	.251 n.s.
*N*_m_	0.46		6.27		9.31	

Abbreviation: ns, not significant.

^a^ Clusters inferred by SplitsTree and BEAST analyses.

^b^ Populations defined by geographic regions, that is, Eastern Canada, Western Canada, Western US.

^c^ Three populations compared: Bromeae, Triticeae (noncrops), and crops in Triticeae.

^d^ Molecular variance among and within populations, *Φ*_PT_ (analog of *F*_ST_), were estimated using GenAlEx; *S*_nn_ (Hudson, 2000) and *N*_m_ (Nei, 1982) estimates of gene flow (migration number per generation) were from dnaSP.

* .01 < *p*<.05; **.001 < *p*<.01; ***p < .001.

**FIGURE 5:**
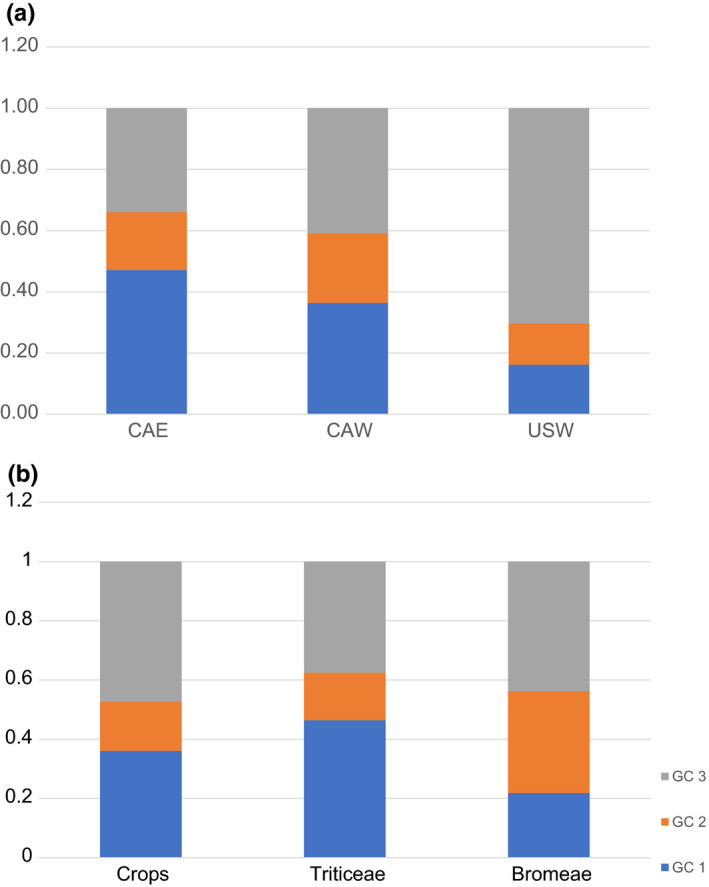
Percentages of three genetic clusters in three geographic regions (a), and three host groups (b)

**FIGURE 6:**
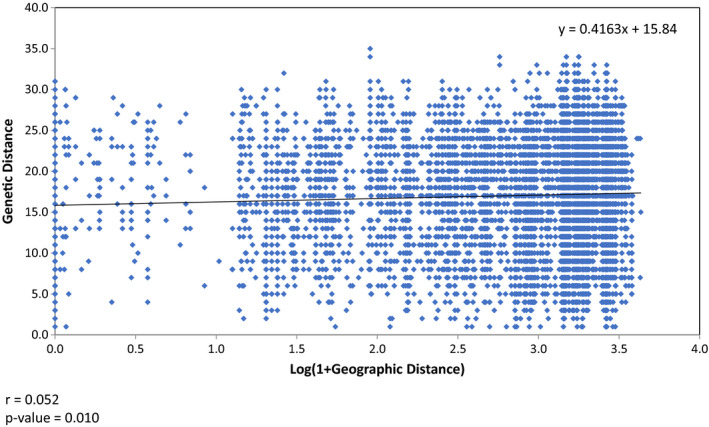
Mantel test for isolation by distance (IBD) showing no evidence of a correlation between genetic distance versus log geographic distance

The pairwise comparison of genetic differentiation between genetic clusters, geographic regions, and host groups showed that three genetic clusters were significantly differentiated from each other, and the values of Φ_PH_ and Nei's D between GC1 and GC3 were the greatest (0.51, 0.088). Pairwise comparisons between Western U.S. and Canadian regions (CAE, CAW) were also statistically significant; however, the level of the differentiation as estimated by Φ_PH_ values and Nei's D was an order of magnitude lower than those between genetic clusters (Table 4). The differentiation between populations associated with host groups Bromeae and Triticeae was close to being significant (Φ_PH_ = 0.023, *p*‐value .056) and was not significant between crops and each grass tribe (Table 4). These patterns can also be observed by examining the percentage of GCs in each geographic region and host group. In western US region, which is comprised of intermountain regions in Colorado and Wyoming, isolates belong to the genetic cluster GC3 were the most abundant, composing 70% of the *C. purpurea* isolates recovered. The GC3 genetic cluster was also the most frequently observed population in western Canada (41%) (Figure 5a). However, GC1 was the most abundant population in eastern Canada, comprising 47% of the isolates recovered (Figure 5a). In all region, GC2 was the least abundant genetic cluster observed, never representing more than 22% of *C. purpurea* isolates recovered (Figure 5a). Mantel test on isolation by distance revealed a low correlation between genetic distances (among the haplotypes) and the logarithm of geographic distances, with very low value of *r* (0.052), however, *p*‐value = .010, indicating significant nonrandom distribution of haplotypes (Figure 6). For host groups, the Crops (Triticeae) and Bromeae groups had higher GC3 (47%, 44% respectively) than GC1 (36%, 22%), while Triticeae (noncrops) groups had a higher percentage of GC1 genotype (46%) than GC3 (38%) (Figure 5b). Bromeae group had a higher percentage of GC2 (34%) genotype than the other two groups.

**TABLE 4:** Pairwise genetic differentiation measured as Φ_PT_(analogous of *F*_ST_), and *N*_ei_ genetic distance between genetic clusters, geographic populations, and host groups

	Genetic clusters				Geographic regions				Host groups		
	GC 1	GC 2	GC 3		CAE	CAW	USW		Crops	Triticeae	Bromeae
GC 1		0.057	0.088	CAE		0.003	0.012	Crops		0.003	0.004
GC 2	0.38***		0.040	CAW	0.007^ns^		0.008	Triticeae	0.001^ns^		0.006
GC 3	0.51***	0.34***		USW	0.07***	0.044***		Bromeae	0.008^ns^	0.023*	

Note

Upper diagonal listed *N*_ei_ genetic distances, below diagonal Φ_PT_ Values, *p*‐value by 9,999 permutation test ***<.01, **<.05, *<.1, ns > .1

### 3.4 Demographics of genetic clusters

**FIGURE 7:**
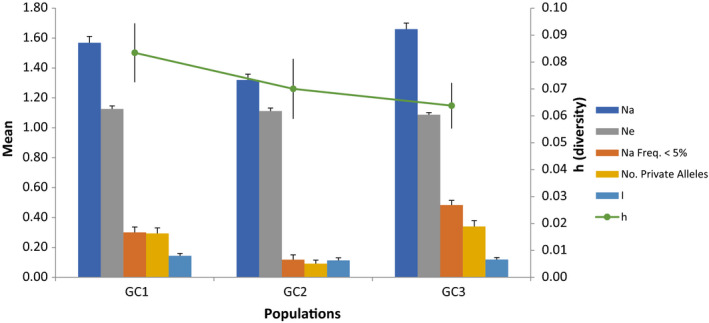
Allelic pattern across three genetic clusters. *N*_a_, number of different alleles; *N*_e_, number of effective alleles = $1/(\sum P_{i}^{2})$, I Shannon's information index = −1×∑(P_i_Ln(P_i_)), *h*, haploid genetic diversity = $1‐\sum P_{i}^{2}$, where *P*_i_ is the frequency of the *i*th allele for the population (cluster)

Allelic analyses of haplotypes showed that GC1 had a slightly higher number of effective alleles *N*_e_ = 1.13 than GC2 (1.11) and GC3 (1.09), highest allelic diversity measured as Shannon's information index *I* = 0.144, and haploid genetic diversity *h* = 0.083. GC3 had highest total allele number (*N*_a_ = 1.66), rare (frequency < 5%), and private allele frequencies (0.48, 0.34; Figure 7). Analyses of DNA sequences of 156 samples in DnaSP v6 showed that GC1 had slightly higher nucleotide diversity (*π* = 0.00555), while GC3 had higher mutation rates per site and per sequence (*θ *− *w* = 0.0083, *Θ *− *W* = 19.450). Neutrality tests based on Tajima's D, Fu, and Li's D* and F* all suggested GC3 was under significant selective pressure. GC1 was under detectable pressure based on Fu and Li's D* (*p*‐value < .1), but not based on Tajima's D and Fu and Li's F*. GC2 was not under selection based on all parameters (Table 5). Two of the recombination parameters of the combined population of all three populations (*R*_g_ = 61.5, *R*_a_ = 0.0262) were lower than the three genetic clusters independently. These parameters were the highest in GC2 (176, 0.0751), further supporting population subdivision. Combination of GC1 and GC2 resulted in lower values of *R*_g_ (109) and *R*_a_ (0.0465) compared with each individual genetic clusters (GC1: *R*_g_ = 112, *R*_a_ = 0.0478; GC2: *R*_g_ = 176, *R*_a_ = 0.0751). The same pattern was shown when GC1 and GC3 were combined (Table 6). This pattern can be interpreted as the result of the reduced random mating between GC1 and GC2, and between GC1 and GC3. However, when GC2 and GC3 were combined, intermediate values of R_g_ (82) and *R*_a_ (0.035) were resulted, which is consistent with the result of PCoA analyses showing the incomplete separation between GC2 and GC3 (Figure 4a). The estimated minimum recombination events in the combined populations (*R*_m_ = 13–18) were higher than the individual GCs (9–14). A likely explanation could be that *R*_m_ was affected by the sample sizes in that the combined population had a larger sample size (*N* = 85–156 samples) than three GCs (*N* = 55, 30, 71 samples, respectively). The estimates of LD parameters, *Z*_ns_, *Z*_a_, ZZ, Wall's B, and Q, were very low (<0.1, or ≈ 0) suggesting frequent recombination in GCs and among GCs in general. In comparison with individual GCs, the lower values of combined populations, that is, GC1 with GC2, and GC1 with GC3, could suggest the lower recombination during selective sweeping between GC1 and GC2 and between GC1 and GC3 (Kelly, 1997).

**TABLE 5:** Genetic diversity and neutrality of genetic clusters based on four genes concatenated alignment

Subpopulation	Genetic diversity					Neutrality					
	Number of sequences	Number of haplotypes	Nucleotide diversity	Theta (per site) from S^a^	Theta (per sequence) from S	Tajima's *D*	*p*‐value	Fu & Li's D*	*p*‐value	Fu & Li's F*	*p*‐value
	*N*	*h*	π ± *SD*	θ − *W* ± *SD*	Θ‐W	Tjm *D*	P	Fu & Li D*	P	Fu & Li F*	P
GC 1	55	53	0.0056 ± 0.0003	0.0079 ± 0.0009	18.577	−1.1285	>.10 ns	−1.8955	<.1*	−1.9198	>.1 ns
GC 2	30	29	0.0048 ± 0.0002	0.0056 ± 0.0008	13.126	−0.5919	>.10 ns	−0.9500	>.1 ns	−0.9825	>0.1 ns
GC 3	71	64	0.0041 ± 0.0002	0.0084 ± 0.0009	19.45	−1.7398	<.1*	−4.0161	<.02***	−3.7277	<.02***
Combined	156	146	0.0072 ± 0.0124	0.0116 ± 0.0009	27.205	−1.326	>.10 ns	−4.8238	<.02***	−3.8362	<.02***

**TABLE 6:** Recombination and linkage disequilibrium between genetic clusters based on four genes concatenated alignment

GCs	Recombination				Linkage disequilibrium				
	*N*	*R*_g_	*R*_a_	*R*_m_	*Z*_nS_	*Z*_a_	ZZ	Wall's B	Wall's Q
GC 1	55	112	0.0478	14	0.0366	0.0979	0.0613	0.0741	0.0613
GC 2	30	176	0.0751	11	0.0501	0.103	0.0529	0.04	0.0588
GC 3	71	65.8	0.0281	9	0.0293	0.1624	0.1331	0.1209	0.1848
GC1 + GC2	85	109	0.0465	16	0.026	0.0597	0.0337	0.0288	0.0571
GC1 + GC3	126	35.8	0.0153	17	0.0216	0.0817	0.0602	0.0469	0.0698
GC2 + GC3	101	82	0.035	13	0.0197	0.102	0.0823	0.0811	0.1339
All	156	61.5	0.0262	18	0.0163	0.0638	0.0475	0.0352	0.049

## 4 DISCUSSION

Our study is the first in‐depth investigation of the population structure of *C. purpurea* s.s (excluding previously identified phylogenetic species) in Canada and western USA using multilocus genotyping. The results from the network analyses (Figure 3), AMOVA (Table 3), PCoA (Figure 4), and demographic parameters (Hudson's *S*_nn_, Nei's genetic distance and *N*_m_; Tables 3 and 4) clearly demonstrated the existence of three genetic clusters (GC1–3; Figure 3) that co‐exist throughout Canada and western U.S. on agricultural and nonagricultural grass species.

Coalescence analyses suggested a relatively recent divergence of GC3 from GC2, whereas the divergence between GC1 and GC2 was more ancestral (Figure 3b). This evolutionary trajectory was supported by the rooted phylogeny (Figure S2) and by demographic parameters; that is, GC1 and GC2 had higher estimates of effective allele number, allele richness (Shannon's information index), haploid diversity, and nucleotide diversity than GC3 (Figure 7, Table 5). Moreover, GC3 had higher mutation rates (*θ* − *w* = 0.0084, *Θ* − *w* = 19.35), higher rare, and private alleles and showed the deviation from neutrality, indicating GC3 is likely experiencing recent fast adaptation under selective pressure. The separation of three genetic clusters seems consistent with what was found in Oregon and Washington using simple sequence repeat markers (Gilmore et al., 2016). In that study, four groups were recovered: Group 1 was distantly related to other three groups, and later determined to be a different species, *C. humidiphila* (Pažoutová et al., 2015). Groups 2 and 3 were more closely related to each other than to group 4. It is likely that group 4 identified by Gilmore et al. (2016) corresponds to GC1 in this study, and the other two groups correspond with GC 2 and GC3 in our study.

High levels of genetic variation and a high proportion of private and rare haplotypes in each genetic cluster suggest a rapid expansion and disruptive selection after introduction or genetic drift (bottleneck) and limited gene flow between the genetic clusters (Milgroom, 2015b). Since neither geographic location nor host range seem to be creating barriers among the three GCs, some other evolutionary force must be maintaining the separate populations. Sexual or vegetative incompatibility, another potential mechanism, might be maintaining this division. Reduced recombination between GC1 with either GC2 or GC3 was demonstrated by the comparison of estimates for recombination rates (*R*_g_ and *R*_a_) and LD (*Z*_ns_, *Z*_a_ and ZZ) within and between the GCs (Table 6). In addition, during a sclerotium germination experiment for a companion study, Liu et al. (2020) observed an interesting phenomenon that raised several questions regarding sexual reproduction in *Claviceps* species. Several sclerotia of *C. ripicola* (a close relative of *C. purpurea*), after chill treatment and incubation for 8–10 weeks, produced tiny buds on sclerotia, and then, these buds stopped growing up to normal stromata (Liu et al., 2020). The authors speculate that the abortion might be due to the absence of a compatible partner. Although Esser and Tudzynski (1978) demonstrated that heterokaryosis is not required for the completion of the life cycle in *C. purpurea*, other studies reported heterokaryosis occurred frequently on sclerotia and occasionally in artificial media (Amici et al., 1967; Tudzynski, 2006). Mating between different strains can be obtained by inoculating rye florets with mixed conidial suspensions (Tudzynski et al., 1982). If homothallism is the most common state for *C. purpurea* s.s., this would also help to maintain the three distinct genetic clusters. The occasional outcrossing may result in novel lineages, perhaps even GC3. However, an in‐depth understanding of genetic mechanisms in *Claviceps* is lacking. Vegetative compatibility has been applied for intraspecific classification of many sexual and asexual fungal species, including species closely related to ergot fungi, that is, *Epichloë* spp. (Chung & Schardl, 1997; Leslie, 1993), but has been understudied in *Claviceps*. The modest progress in genetic studies in *Claviceps* has been attributed to the technical challenges (Tudzynski, 2006), including long generation time, complex conditions for obtaining sexual progeny, unstable asexual proliferation, and frequent degeneration of vegetative growth. However, PCR‐based assays could be optimized for testing large sample sizes (Yokoyama et al., 2004), and high‐throughput techniques in recognizing vegetative compatible groups could also help overcome some of these obstacles and improve our understanding of genetic recombination in *Claviceps purpurea* (Papaioannou & Typas, 2015; Salman et al., 2015). Understanding the mating systems and vegetative compatibility in *C. purpuea* populations would help to identify reproduction barriers and shed light on the mechanisms of population subdivision in *Claviceps purpurea*.

The genomes of the members in three GCs may also be evolving and adapting to other environmental pressures, resulting in subpopulations remaining separated. A recent comparative genomic analysis of the genus *Claviceps* found that species in the section *Claviceps*, such as *C. purpurea*, have adaptive genomes through colocalization of transposable elements around predicted effectors and a putative loss of repeat‐induced point mutation (Wyka, Mondo, Liu, Dettman, et al., 2020). This has resulted in unconstrained tandem gene duplication coinciding with increased host range potential and speciation (Wyka, Mondo, Liu, Dettman, et al., 2020). These alterations in genomic architecture and plasticity can influence and shape the evolutionary trajectory of fungal pathogens and their adaptability. Members of the genus *Claviceps* are renowned for their production of secondary metabolites, which may serve to improve overall fitness of the organism. A pangenome analysis of 24 genomes found that *C. purpurea* has a relatively large accessory genome (~38%) that is likely maintained by high recombination rates and transposon‐mediated gene duplication, but the high recombination rate is also likely influencing the overall trend of purifying selection across the genome (Wyka et al., 2020). This purifying or stabilizing selection may be purging deleterious genetic polymorphisms that arise from random mutations and transposon‐mediated gene duplication. However, Wyka, Mondo, Liu, Dettman, et al., 2020 did observed evidence of strong positive selection pressure on secondary metabolite genes and that the *lpsA1* and *lpsA2* (genes in the ergotamine synthesis pathway) were the results of a recombination event. It is possible that the combination of positive selection on secondary metabolite genes with purifying selection across the rest of the genome has resulted in a more specific, as yet undetected, niche adaptation followed by population stabilization that has resulted in the observed patterns in geographic and host overlap of the three GCs found in North America.

### 4.1 The neutrality of two house‐keeping genes (*EF1*‐α and *RPB2*)

The initial neutrality tests for each individual gene indicated only *easA* was not significantly deviating from neutral, while the other three genes all showed significant deviation at varied critical levels (Table 1). This is unexpected as it was generally accepted that house‐keeping genes; that is, *TEF1*‐α and *RPB*2 are neutral. Structured population could account for biased estimates of neutrality parameters; therefore, we conducted the tests for separate GCs. There was no significant deviation in *TEF*1‐α and *easE* in GC1 and GC2, but significant deviation from neutrality in GC3 for all three parameters. *RPB*2 was neutral in all three GCs with Tajima'D, but deviated significantly at 0.05 critical level with Fu and Li's D* and F*, which are more sensitive tests based on coalescence approach. Overall, it appears that *RPB*2 is under moderate selection pressure in all populations, while *TEF1*‐α and *easE* are under strong selection only in GC3, which might be associated with the divergence of GC3. Understandably, *easE* is experiencing positive selection (as inferred by a negative value of Tajima'D) because it is common that genes involving secondary metabolite production are under selective pressure (Wyka, Mondo, Liu, Dettman, et al., 2020). The observed high level of genetic variation (polymorphisms, nucleotide, and haplotype diversity) is consistent with the scenario of positive selection. Compared with *easE*, *TEF1*‐α and *RPB*2 are much more conserved. A likely explanation is that *TEF*1‐α and *RPB2* are linked with genes or regions under positive selection and have undergone “genetic hitchhiking,” or what is referred to as “selective sweeping.” In this case, the selective pressure on the region linked with *TEF1*‐α is higher than on the regions linked with *RPB2*. Both *TEF1‐α* and *RPB2* genes have been widely used in phylogenetics and species barcoding of various fungal groups because of many advantageous features including that they are selectively neutral (Brandon Matheny et al., 2007; Geiser et al., 2004; O'Donnell et al., 2009). Our results challenge this assumption. The situation may vary in different fungal groups and may or may not always have a significant impact on the species level studied. In *Claviceps*, some cryptic species noticeably separated based on RPB2 sequence data but showed very little variation in *TEF1*, and vice versa (Liu et al., 2020; Shoukouhi et al., 2019). A holistic approach is recommended to overcome the bias caused by either one of these genes. As these two genes are being considered as a universal secondary fungal barcoding region, perhaps both genes should be considered.

## CONFLICT OF INTEREST

No conflict.

## AUTHOR CONTRIBUTION

**Miao Liu:** Data curation (supporting); Formal analysis (lead); Funding acquisition (equal); Investigation (equal); Methodology (supporting); Project administration (lead); Resources (supporting); Supervision (lead); Validation (equal); Visualization (equal); Writing‐original draft (lead); Writing‐review & editing (equal). **Parivash Shoukouhi:** Data curation (lead); Investigation (equal); Methodology (lead); Resources (supporting); Supervision (supporting); Writing‐review & editing (supporting). **Kassandra Bisson:** Data curation (lead); Investigation (equal); Methodology (equal); Resources (supporting); Writing‐review & editing (supporting). **Stephen A. Wyka:** Data curation (lead); Investigation (equal); Methodology (equal); Resources (equal); Writing‐review & editing (equal). **Kirk D. Broders:** Data curation (supporting); Funding acquisition (equal); Project administration (equal); Supervision (equal); Validation (equal); Writing‐original draft (supporting); Writing‐review & editing (equal). **Jim G. Menzies:** Data curation (supporting); Funding acquisition (supporting); Investigation (equal); Methodology (equal); Project administration (supporting); Resources (lead); Supervision (equal); Writing‐review & editing (supporting).

## Supporting information

Fig S1

Fig S2

Table S1

Table S2

## Data Availability

All DNA sequences were submitted to GenBank (Table S1). GB accessions: *TEF*1‐α MT429981–MT430207, RPB2 MT429777–MT429980, *easE* MT430208–MT430471, *easA* MT430472–MT430745.
