# Supplementary figures and images for: Sympatric divergence of the ergot fungus, Claviceps purpurea, populations infecting agricultural and nonagricultural grasses in North America
Source: Ecol Evol. 2020 Dec 12;11(1):273–93. doi: 10.1002/ece3.7028 (PMC7790621; doi:10.1002/ece3.7028)

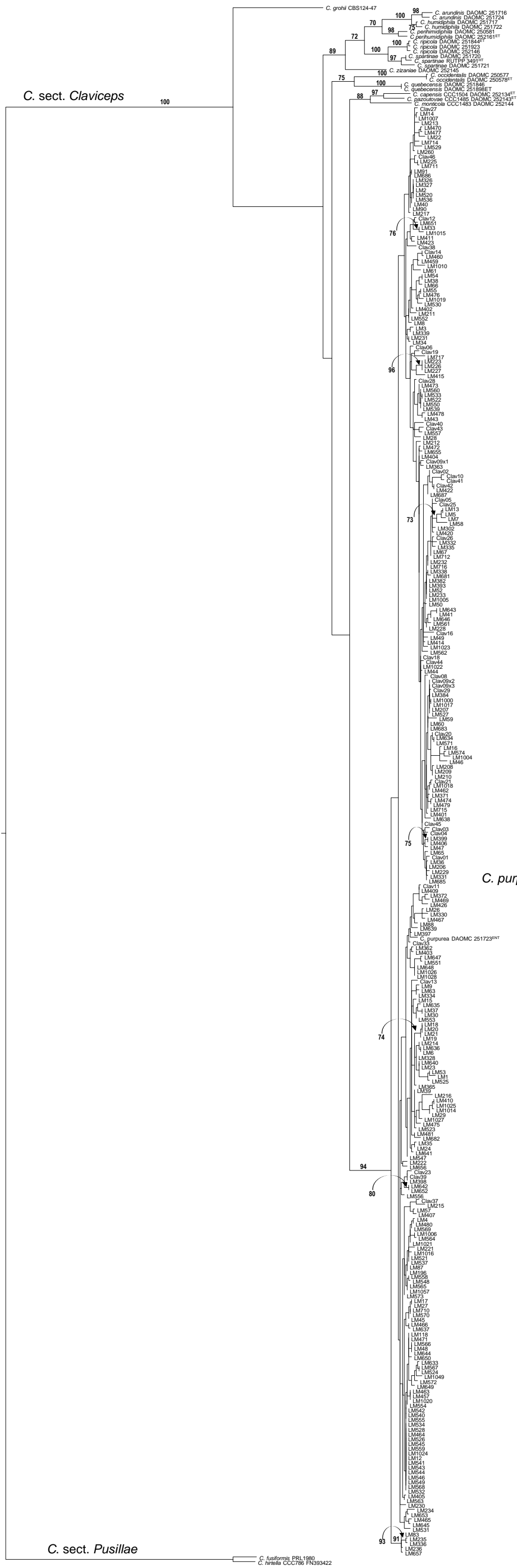

Supplementary Figure 1

Supplement: Supplementary file 1 — Fig S1 [file ECE3-11-273-s001.pdf]

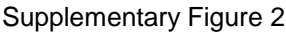

Supplement: Supplementary file 2 — Fig S2 [file ECE3-11-273-s002.pdf]
